# Social Networking Addiction Among Hong Kong University Students: Its Health Consequences and Relationships With Parenting Behaviors

**DOI:** 10.3389/fpubh.2020.555990

**Published:** 2021-01-25

**Authors:** Lu Yu, Tingyu Luo

**Affiliations:** ^1^Department of Applied Social Sciences, The Hong Kong Polytechnic University, Hong Kong, Hong Kong; ^2^Department of Social Work and Social Administration, The University of Hong Kong, Hong Kong, Hong Kong

**Keywords:** social networking addiction, social networking site, parenting, well-being, university students, Hong Kong

## Abstract

The use of social networking sites (SNSs) has been growing at a staggering rate, especially among university students. The present study investigated the prevalence of social networking addiction (SNA), its health consequences, and its relationships with parents' Internet-specific parenting behaviors in a sample of Hong Kong university students (*N* = 390). Adopting the 9-item social media disorder scale, 21.5% of the participating students met the criteria for SNA. Students with SNA showed longer sleeping latency, more sleep disturbance, poorer academic performance, lower levels of life satisfaction, and higher levels of depression than did students without SNA. Parental reactive restriction and limiting online behaviors of the participants were associated with higher risk of SNA. The findings suggest the severity of SNA and its negative consequences among Hong Kong university students. While parental behaviors limiting children's use of SNSs were found to increase the occurrence rate of SNA among university students, longitudinal studies are needed to further examine this causal relationship.

## Introduction

Social networking sites (SNSs) are defined as web-based services that allow users to “(1) construct a public or semi-public profile within a bounded system, (2) articulate a list of other users with whom they share a connection, and (3) view and traverse their list of connections and those made by others within the system.” (p. 211) (1). Through SNSs, people can easily communicate and interact with their friends, family, and people from all over the world on the Web. Facebook, Twitter, and Instagram are among the most popular SNSs while new platforms/websites continue to pop up regularly. A recent report showed that in 2019, there were 3.48 billion SNSs users worldwide (almost half of the Earth's entire population), of which 27% were young adults aged between 18 and 24 years old (2). Internet users also reported that approximately 27% of the time spent online was for social media interaction, more than for email, news, and any form of entertainment combined (3). Using SNSs has become not only the most popular online activity but an important part of our daily lives (4).

While SNSs have brought us enormous convenience and enjoyment, researchers have observed a compulsive tendency among a proportion of SNSs users (5–7). These users feel compelled to use SNSs more excessively to maintain their online social networks, and they also tend to show symptoms that are traditionally associated with substance or other behavioral addiction, such as salience, withdrawal, relapse, growing tolerance, and mood modification (8). This phenomenon has been termed social networking addiction (SNA) or social media addiction, defined as the status of “being overly concerned about SNSs, driven by a strong motivation to log on to or use SNSs, and to devote so much time and effort to SNSs that it impairs other social activities, studies/job, interpersonal relationships, and/or psychological health and well-being” (p. 4,054) (9).

Although SNA has not been formally recognized as a diagnosis, empirical findings across the world generally show that the overall prevalence of SNA is not low. For example, based on a systematic review, Andreassen (10) reported that the prevalence rates of SNA ranged between 1.6% (in a Nigerian sample) and 34% (in a Chinese student sample). Growing evidence suggests that SNA is associated with various adverse consequences, such as: poor sleep quality (11), reduced academic and work performance (12), relationship problems (13), impaired self-esteem and life satisfaction (14), and mental health problems (15, 16). Researchers have also found brain anatomy alterations associated with SNA, including reduced gray matter volumes in the amygdala bilaterally (17), that are similar to those associated with other forms of behavioral addiction (e.g., gambling).

It is worth noting that university students may be particularly vulnerable to SNA for several reasons. First, university students have notably high Internet literacy which enables them to be the predominant users of SNSs (18, 19). Second, compared with secondary school students, university students' online activities are less supervised by their teachers and parents. Third, university students typically have flexible schedules and more free and unlimited access to SNSs. Fourth, developmental characteristics associated with youth may also increase the attraction of SNSs for university students. SNSs use offers young adults new and convenient opportunities to interact with different people, build up intimate relationships, and further develop their identities (20). In fact, ample evidence has been published on the high prevalence of SNA among university students. For instance, using an adapted 6-item Bergen Facebook Addiction Scale (BFAS) (8), Tang and Koh (21) surveyed 1,100 Singapore college students and found that 29.5% of the respondents could be categorized as having SNA. Another study in China based on Young's Internet addiction criteria revealed an SNA prevalence of 34% among Chinese college students. In a recent review, it was further revealed that greater exposure to online social networks was associated with greater alcohol use and other addictive behaviors among college students (22). This suggests that SNA in university students has become an important public health issue which deserves more attention from both researchers and the public (4).

Hong Kong is among one of the areas with the highest social media penetration rate in the world. According to the Census and Statistics Department of the Hong Kong Government (23), 75% of the population are active SNS users, with young adults constituting the majority. It is estimated that the number of young SNS users will continue to grow as more attractive SNS applications and platforms become available. However, direct evidence regarding the prevalence of SNA among Hong Kong university students is limited. Existing studies have either focused on the generalized form of Internet/mobile phone addiction (24, 25), or only examined the use of a specific SNS such as Facebook (26). Addictive use of other popular SNSs (e.g., WhatsApp, WeChat, and Twitter) have not been considered, which may mean that the reported prevalence of SNA is an underestimate. Moreover, the consequences of SNA on different domains of quality of life (i.e., physical, mental, and academic) have not been systematically evaluated. Therefore, much remains unknown about how severe SNA is among Hong Kong university students and its impact on students' well-being.

To prevent SNA in university students, it is important to identify the risk and protective factors in the development of SNA. Past research has revealed that specific personality traits (e.g., narcissism, neuroticism), psychological characteristics (e.g., self-esteem), and social factors (e.g., peer influence, loneliness) contribute to the formation of SNA (27, 28). However, the role of family in the development of SNA among university students has received little attention, although the impact of parenting behaviors on adolescents' Internet addiction has been reported widely (29–31). For example, neglecting parenting style, inconsistencies, and contradictions between parents (32), family conflicts (33), and parents' psychological control (34) have been found to be associated with increased risk of developing Internet addiction. Parental warmth and acceptance (35), behavioral control (36, 37), and good parent-child relationship (38) had negative associations with Internet addiction. Apart from the effects of general parenting style and family functioning, studies have reported on the role of Internet-specific parenting practices, referred to as parenting behaviors that specifically aim to guide their children's use of the Internet and minimize its negative consequences (39), in protecting youth from Internet addiction (33, 40). For instance, Chng et al. (41) found that parental restrictive mediation (i.e., parents' restriction of their children's Internet use) was negatively related to adolescents' Internet addiction based on a sample of Singapore secondary school students. In van den Eijnden et al.'s (39) study, parental active mediation, defined as interaction and communication regarding online activities between children and parents, predicted decreased pathological Internet use. Nevertheless, few studies investigating how parenting behaviors may influence university students' addictive behaviors including SNA have been published.

This lack of research could be explained by the fact that the increased autonomy of university students results in their declined reliance on family in their development (42). They attempt to separate their lives from the control of their parents to develop independence and take responsibility of their own world (43). Thus, the effects of parenting behaviors are considered as collectively less important than those of other factors, such as peers and personal attributes. Nonetheless, a few studies suggest that parents continue to exert direct and indirect effects on young adults' lives, whether in the psychological or social domain. For instance, researchers have reported significant relationships between university students' substance use and their parents' behavioral control, family communication, and parent-child relationship (44–48). Parental rejection and paternal overprotection have been found to be positively associated with Chinese university students' risks of Internet addiction (49).

In the context of Hong Kong, the influence of parents on university students may be more profound for several reasons. First, due to the limited residential places offered by universities and high housing expenses, most Hong Kong local students remain living with their family members after entering university (50). This potentially makes the influence of parenting behaviors greater on university students in Hong Kong than in other countries and areas. Second, parental influence on young people's behaviors may also be strengthened by Chinese cultural values, which emphasize parental authority and children's obedience (51). Third, although Chinese parents are likely to be more restrictive than Western parents (52), Chinese children tend to consider their parents' behavioral monitoring as a sign of parental warmth and responsiveness (53–55). Thus, it is possible that Hong Kong university students may be more receptive to their parents' mediation regarding their Internet use than people in cultures where autonomy is more emphasized. Based on these socio-cultural characteristics, it seems reasonable to assume that parents of Hong Kong university students may still play an influencing role in their development of SNA.

### The Present Study

Considering the background explained above, the present study had three purposes. First, we aimed to preliminarily examine the prevalence of SNA based on a sample of Hong Kong university students. Second, we sought to investigate the association between SNA and a variety of health outcomes, including sleep quality, depression, life satisfaction, and academic performance among Hong Kong university students. Third, we intended to address the predictive effects of parenting behaviors on university students' SNA. In particular, we focused on Internet-specific parenting practices based on the parental mediation theory (56). According to this theory, three types of parenting practices are related to youth compulsive SNS use: active mediation (i.e., parent-adolescent conversations regarding online activities), restrictive mediation (i.e., allowance to use particular online applications), and social co-use (i.e., children using the Internet in the company of their parents, such as watching movies online and playing Internet games together). Apart from parental mediation, parents' own use of SNSs is regarded as another factor that influences their children's behaviors through social modeling. Previous studies have supported the protective effect of these parenting practices on adolescents' problematic use of computers, electronic devices, and the Internet (39, 57). In the present study, the respective effects of the three types of parenting behaviors on university students' SNA were investigated.

## Methods

### Participants and Procedure

The present study was carried out with undergraduate students in a public university in Hong Kong. In 2018/19 academic year, students taking a selective introductory psychology course offered to all undergraduate students in the university were recruited on a voluntary basis. An invitation letter containing a brief introduction about the study and a consent form was emailed to all students enrolled in this subject (*N* = 490 including 217 male students and 273 female students). Students who agreed to participate were required to return their signed consent forms, after which they were provided with a link to an online questionnaire. They completed the survey anonymously within the first month of the semester. Ethical approval of the study was obtained from the Human Subjects Ethics Committee of the first authors' institution. All data collected in the study were kept strictly confidential and analyzed in an aggregated manner.

The final sample of participants totaled 390 students (Mean age = 19.09 years, SD = 1.47) consisted of 176 (45.13%) males and 214 (54.87%) females, indicating an overall response rate of 79.6%. The students were from eight different faculties and schools of the university, with the majority from the School of Business (*n* = 246, 63.07%) followed by students in the Faculty of Health and Social Sciences (*n* = 54, 13.85%). Regarding year of study, 68.5% of the participants (*n* = 267) were in their first year of university, 18.7% (*n* = 73) were in their second year, 6.4% (*n* = 25) were in their third year, 5.6% (*n* = 22) were in their fourth year, and 0.8% (*n* = 3) were in their fifth year.

### Measures

To measure participants' SNS usage behaviors, SNA, mental health status, and their parents' Internet-specific parenting behaviors, five core scales were used, which are described below. Cronbach's alpha of each scale for the present sample is presented in Table 1. Apart from these scales, students responded to a few items asking about their demographic information and academic performance in the latest semester. Specifically, we asked students to report their perceived academic performance, comparing to other students of the same cohort, in the last semester, based on a five-point rating scale (1 = very poor, 2 = poorer than others, 3 = average, 4 = better than others, and 5 = very good).

**Table 1:** Descriptive statistics of the key variables.

**Continuous variables**	**Total (*****N*** **=** **390)**			**Male (*****N*** **=** **176)**		**Female (*****N*** **=** **214)**		
	**Cronbach's alpha**	**Mean**	**SD**	**Mean**	**SD**	**Mean**	**SD**	***t***
Age	–	19.09	1.47	19.11	1.79	19.04	1.14	0.45
Life satisfaction	0.86	4.10	1.16	4.03	1.14	4.16	1.18	1.15
Depression	0.87	1.95	0.54	1.98	0.51	1.93	0.57	−0.90
Sleep disturbance	0.81	0.65	0.54	0.71	0.53	0.61	0.54	−1.88
Sleep latency	–	1.29	1.05	1.40	1.06	1.20	1.04	−1.84
Perceived academic performance	–	3.00	0.75	2.91	0.83	3.07	0.67	2.12
Specific parenting behaviors								
Reactive restriction	0.88	1.71	0.82	1.90	0.89	1.54	0.72	−4.32^***^
Internet-specific rules	0.92	3.24	0.99	3.22	0.97	3.25	1.01	0.39
Limiting online behavior	0.77	1.97	0.77	2.10	0.80	1.86	0.73	−3.09^**^
Communication about the internet	0.74	3.09	0.92	3.01	0.90	3.16	0.93	1.55
Co-use	0.84	2.25	0.89	2.17	0.86	2.31	0.92	1.55
Parent-adherence	0.82	2.47	0.82	2.54	0.83	2.41	0.81	−1.54
SNA	0.72	2.80	2.21	2.96	2.36	2.66	2.08	−1.30

** p < 0.01;

*** *p < 0.001*.

#### Social Media Disorder Scale (SMD)

In the present study, we adopted the 9-item Social Media Disorder Scale (58) to assess participants' SNA. The SMD was developed using similar diagnostic criteria to that of Internet Gaming Disorder (DSM-V). Each item measures a unique dimension of SNA, including preoccupation, tolerance, withdrawal, persistence, escape, problems, deception, displacement, and conflict. Respondents answer “Yes” or “No” regarding whether they have had the listed SNA behavior in the past 1 year. Example items include “often used social media to escape from negative feelings,” and “tried to spend less time on social media, but failed.” A person is classified as having SNA if he or she shows five or more of the listed behaviors. Previous studies have demonstrated the robust psychometric properties of the 9-item SMD (59, 60). Cronbach's alpha of this scale for the current sample was 0.72.

#### Satisfaction With Life Scale (SWLS)

Life Satisfaction was assessed using the 5-item Satisfaction with Life Scale (61), a well-known self-reported scale. Each item is rated on a 7-point Likert scale ranging from 1 (strongly disagree) to 5 (strongly agree). A higher score represents a higher level of life satisfaction. The SWLS has shown good psychometric properties in different cultural contexts with different populations (14, 62, 63). In the present study, the scale had a high Cronbach's alpha of 0.86.

#### Patient Health Questionnaire (PHQ-9)

Depression was measured using the 9-item Patient Health Questionnaire (64), a self-report tool developed to assess mental disorder using DSM-IV criteria. The PHQ-9 has been translated into various languages and evaluated in different ethnic groups and countries, including China (65, 66). Participants rated each item based on a 4-point Likert scale (0 = not at all; 3 = nearly every day). A higher score represents a higher level of depression. The Cronbach's alpha for the current sample was 0.87.

#### Internet-Specific Parenting Behaviors Scale

Internet-specific parenting behaviors were assessed using Koning et al.'s (57) scale adapted from an earlier version measuring parenting behaviors related to Internet use developed by van Den Eijnden et al. (39). The adapted scale asks participants to report the frequency of certain behaviors displayed by their parents when they want to be online (i.e., everything they do on the Internet with a smartphone, tablet, or computer) or gaming, including six subscales that measure three major types of parental mediation (restrictive mediation, active mediation, and social co-use) and parents' own use of SNSs. “Reactive restriction” (4 items) reflects parents' restrictive responses to their children's use of SNSs or gaming (e.g., “how often do your parents say that you can't be on social media or gaming?'). “Internet-specific rules” (9 items) measures the extent to which parents set strict rules on when and how long their children can use the Internet. The subscale of “limiting online behaviors” (4 items) assesses parents' controlling behaviors that stop children from using the Internet (e.g., taking away phones or tablets, turning off Wi-Fi, and commanding angrily). These three subscales reflect parents' restrictive mediation. “Quality of communication” (3 items) asks participants about their feelings when their parents talk with them about their online activities, which reflects parents' active mediation. The other two subscales, “co-use” (3 items) and “parent adherence” (4 items), measure the extent to which parents monitor their children's Internet use or remain present while their children are using social media (social co-use), and parents' own use habits at home, respectively. These two subscales show parental modeling and co-using behaviors. Participants responded to each item on a 5-point rating scale, with higher scores representing higher frequency of the measured behaviors. The internal consistencies of the six subscales were all above 0.74 in the present study.

#### Pittsburgh Sleep Quality Index (PSQI)

Participants' sleep quality was measured by nine items excerpted from the Pittsburgh Sleep Quality Index (67). The PSQI is a validated questionnaire that assesses one's quality of sleep and sleep disturbances in the last month (68, 69). Sleep latency was measured by the item “during the past month, how often have you had trouble sleeping because you cannot get to sleep within 30 min.” Sleep disturbance was measured by eight items on different problems that can disturb one's sleep, such as “wake up in the middle of the night or early morning,” and “have to get up to use the bathroom.” Participants rated each item on a 4-point scale from 0 to 3 (0 = not during the past month; 1 = less than once a week; 2 = once or twice a week; 3 = three or more times a week). In this study, Cronbach's alpha for the eight sleep disturbance items was 0.81.

### Statistical Analysis

First, descriptive statistics on participants' problematic use of SNSs and prevalence of SNA were calculated for the whole sample, and for students of different gender, respectively. Independent samples *t*-tests were performed to examine whether males and females differed in the key variables. Bivariate correlation analyses were also conducted to test the preliminary associations between SNA, health indicators, and Internet-specific parenting behaviors. Second, independent-samples *t*-tests were conducted to compare students with and without SNA in terms of their life satisfaction, depression, sleep latency and disturbance, and perceived academic performance. Third, logistic regression was conducted to examine the extent to which different Internet-specific parenting behaviors would predict university students' SNA based on the whole sample, and on samples of males and females, respectively.

### Ethics

The study procedures were carried out in accordance with the guidelines of the Declaration of Helsinki. The data collection received ethical approval from the Human Subjects Ethics Sub-committee of the first author's university. All subjects were informed about the study and all of them signed consent forms before the survey.

## Results

Descriptive statistics of all variables in this study were first calculated and summarized. Table 1 presents the means and standard deviations of all continuous variables (e.g., age, life satisfaction, and depression). For parents' reactive restriction (*t* = −4.32, *p* < 0.001) and limiting online behavior (*t* = −3.09, *p* < 0.01), male students scored significantly higher than female students. All other variables did not show significant gender difference. Table 2 summarizes simple correlation coefficients among SNA, health indicators, and Internet-specific parenting behaviors. Based on the whole sample, the correlations between health indicators and SNA were all significant (*ps* < 0.05). High SNA was associated with longer sleep latency, more sleep disturbance, higher level of depression, lower life satisfaction, and lower perceived academic performance. On the other hand, Internet-specific parenting behaviors including reactive restriction, limiting online behavior, co-use, and parent adherence were positively related to participants' reported SNA. Similar findings were observed in the male sample and the female sample, respectively. For both males and females, SNA was positively related to depression and sleep latency; SNA was also found to be positively associated with parents' reactive restriction, limiting online behavior, and parents' co-use.

**Table 2:** Correlations between social networking addiction, health indicators, and internet-specific parenting behaviors.

**The whole sample^**a**^**	**SNA**	**LS**	**Depression**	**SLD**	**SLL**	**RR**	**IR**	**LB**	**QC**	**CU**	**PA**
SNA	–										
LS	−0.10^*^	–									
Depression	0.33^**^	−0.22^**^	–								
SLD	0.32^**^	−0.15^**^	0.56^**^	–							
SLL	0.15^**^	−0.06	0.27^**^	0.34^**^	–						
RR	0.33^**^	0.00	0.28^**^	0.26^**^	0.04	–					
IR	−0.06	−0.00	0.05	0.10^*^	0.10^*^	−0.24^**^	–				
LB	0.31^**^	0.02	0.29^**^	0.21^**^	0.00	0.58^**^	−0.25^**^	–			
QC	−0.06	0.13^*^	−0.06	−0.04	−0.07	−0.15^**^	0.19^**^	0.01	–		
CU	0.16^**^	0.12^*^	0.14^**^	0.17^**^	−0.03	0.19^**^	0.02	0.36^**^	0.17^**^	–	
PA	0.11^*^	0.03	0.15^**^	0.18^**^	0.00	0.18^**^	0.18^**^	0.25^**^	0.01	0.30^**^	–
By gender^b^	SNA	LS	Depression	SLD	SLL	RR	IR	LB	QC	CU	PA
SNA	–	−0.13	0.30^**^	0.26^**^	0.09	0.34^**^	−0.02	0.25^**^	−0.02	0.14^*^	0.00
LS	−0.06	–	−0.25^**^	−0.18^**^	−0.07	0.01	0.03	0.00	0.14^*^	0.19^**^	0.07
Depression	0.36^**^	−0.17^*^	–	0.57^**^	0.30^**^	0.30^**^	0.02	0.29^**^	−0.08	0.15^*^	0.02
SLD	0.23^**^	−0.11	0.55^**^	–	0.38^**^	0.14^*^	0.13	0.15^*^	0.02	0.17^*^	0.09
SLL	0.13	−0.04	0.21^**^	0.29^**^	–	0.03	0.13	−0.05	−0.02	−0.01	−0.08
RR	0.30^**^	0.02	0.27^**^	0.36^**^	0.01	–	−0.18^**^	0.52^**^	−0.09	0.22^**^	0.08
IR	−0.11	−0.05	0.11	0.04	0.06	−0.30^**^	–	−0.15^*^	0.17^*^	0.09	0.20^**^
LB	0.35^**^	0.03	0.29^**^	0.26^**^	0.02	0.61^**^	−0.36^**^	–	0.08	0.37^**^	0.18^**^
QC	−0.09	0.11	−0.03	−0.10	−0.11	−0.17^*^	0.20^**^	−0.06	–	0.23^**^	0.04
CU	0.21^**^	0.01	0.13	0.18^*^	−0.03	0.21^**^	−0.08	0.39^**^	0.09	–	0.23^**^
PA	0.22^**^	−0.02	0.30^**^	0.28^**^	0.08	0.25^**^	0.16^*^	0.32^**^	−0.01	0.42^**^	–

*LS, life satisfaction; SLD, sleep disturbance; SLL, sleep latency; RR, reactive restriction; IR, internet-specific rules; LB, limiting online behaviors; QC, quality of communication; CU, co-use; PA, parent adherence*.

a *Pearson correlation coefficients calculated based on the whole sample (N = 390)*.

b *Pearson correlation coefficients calculated for males (N = 176) and females (N = 214), respectively; values below the diagonal are for males; values above the diagonal are for females*.

* p < 0.05;

** *p < 0.01*.

### Prevalence of Social Networking Addiction

Table 3 presents the numbers and percentages of participants who displayed different SNA behaviors in the past year measured by the Social Media Disorder Scale. Nearly half (49.5%) of the participants reported that they often used social media to escape from negative feelings. More than 40% reported that they were preoccupied by social media usage (44.1%), tried to spend less time on social media but failed (44.9%), and often felt bad when they could not use social media (40.0%). These results show that addictive behaviors associated with SNS use are quite common among Hong Kong university students. While relatively fewer participants reported interpersonal problems caused by their social media use (16.2% had arguments with others and 15.4% had serious conflicts with family members), more than one-fifth of the participants said that they regularly neglected other activities because they wanted to use social media. Adopting the criteria used by Van den Eijnden et al. (59), 84 participants could be classified as having SNA, indicating an overall prevalence rate of 21.5% among the current sample of university students. Among the 84 identified students, 44 were males and 40 were females; while the prevalence of SNA appeared to be higher among male students (25.0%) than female students (18.7%), the difference did not reach statistical significance (*X*^2^ = 2.27, *p* = 0.13).

**Table 3:** Prevalence of social networking addiction behaviors.

**During the past year, have you…**	**Yes**	
	**Number**	**Percentage (%)**
1. Regularly found that you can't think of anything else but the moment that you will be able to use the social media again?	172	44.1
2. Regularly felt dissatisfied because you wanted to spend more time on social media?	118	30.3
3. Often felt bad when you could not use social media?	156	40.0
4. Tried to spend less time on social media, but failed?	175	44.9
5. Often used social media to escape from negative feelings?	193	49.5
6. Regularly had arguments with others because of your social media use?	63	16.2
7. Regularly lied to your parents or friends about the amount of time you spend on social media?	70	17.9
8. Regularly neglected other activities (e.g., hobbies, sport) because you wanted to use social media?	84	21.5
9. Had serious conflicts with your parent(s) and/or sibling(s) because of your social media use?	60	15.4
Social networking addiction (yes on five or more items)	84	21.5

### Health Consequences of Social Networking Addiction

Results of the independent samples *t-*tests based on bootstrapping (Table 4) show that participants who met the criteria of SNA displayed longer sleep latency, more sleep disturbance, lower life satisfaction, and higher levels of depression compared to those in the non-SNA group. Students with SNA also reported lower levels of perceived academic performance than did those without SNA. All differences are statistically significant (*ps* < 0.05). Students who displayed more SNA behaviors tended to have poorer health status in multiple areas.

**Table 4:** Independent samples *t*-tests comparing health indicators between SNA and non-SNA groups based on 1,000 bootstrap samples.

**Health indicators**	**Non-SNA group (*N* = 306)**	**SNA group (*N* = 84)**	***t***	***p***
Sleep latency	2.23 (1.06)	2.52 (1.01)	−2.31	0.02
Sleep disturbance	1.58 (0.47)	1.91 (0.66)	−4.28	0.00
Academic performance	3.40 (0.75)	2.85 (0.74)	2.11	0.04
Depression	1.86 (0.50)	2.29 (0.57)	−6.85	0.00
Life satisfaction	4.16 (1.16)	3.88 (1.18)	2.00	0.05

### Predictive Effects of Internet-Specific Parenting Behaviors on Social Networking Addiction

Table 5 reports the results of the logistic regression analysis. First, students' year of study and gender failed to predict the occurrence of SNA. Second, two specific parenting behaviors, reactive restriction (OR = 1.78; 95% CI = 1.24–2.55) and limiting online behaviors (OR = 1.72; 95% CI = 1.11–2.65), were found to be associated with higher probability of students being classified as having SNA. The other four parenting behaviors, including setting strict rules on Internet use, quality of communication, co-use, and parent adherence, all failed to predict the occurrence of SNA. Another two logistic regression analyses were performed on the male sample and the female sample, respectively. Consistent with findings generated from the whole sample, parents' limiting online behaviors were positively associated with higher probability of male students being classified as having SNA (OR = 2.13; 95% CI = 1.11–4.09); parents' reactive restriction was positively associated with female students' risk for SNA (OR = 2.44; 95% CI = 1.40–4.25).

**Table 5:** Logistic regression analysis of the prediction of SNA by parenting behaviors.

	**The whole sample**				**Male students**				**Female students**			
**Predictors**	**B**	**SE**	**OR**	**95% CI**	**B**	**SE**	**OR**	**95% CI**	**B**	**SE**	**OR**	**95% CI**
**Block 1**												
Gender	−0.37	0.25	0.70	0.43–1.13	–	–	–	–	–	–	–	–
Year of study	0.03	0.14	1.03	0.79–1.34	−0.07	0.19	0.94	0.65–1.35	0.14	0.20	1.15	0.78–1.69
**Block 2**												
Reactive restriction	0.57	0.19	1.78^**^	1.24–2.55	0.28	0.25	1.33	0.81–2.18	0.89	0.28	2.44^**^	1.40–4.25
Internet-specific rules	0.11	0.16	1.11	0.81–1.53	0.08	0.26	1.08	0.65–1.80	0.16	0.22	1.17	0.77–1.79
Limiting online behaviors	0.54	0.22	1.72^*^	1.11–2.65	0.76	0.33	2.13^*^	1.11–4.09	0.35	0.31	1.42	0.77–2.62
Quality of communication	−0.17	0.16	0.85	0.62–1.17	−0.25	0.25	0.78	0.47–1.28	−0.09	0.22	0.91	0.60–1.39
Co-use	0.23	0.17	1.26	0.90–1.75	0.22	0.26	1.24	0.75–2.07	0.16	0.23	1.18	0.75–1.84
Parental adherence to rules	−0.01	0.19	0.99	0.69–1.43	0.29	0.28	1.33	0.78–2.29	−0.29	0.27	0.75	0.44–1.27
Cox and Snell *R*^2^	0.12				0.15				0.11			

* p < 0.05;

** *p < 0.01*.

## Discussion

The present study investigated the prevalence of SNA, its health consequences, and its relationships with Internet-specific parenting behaviors in a group of Hong Kong university students. More than one-fifth of the participants could be regarded as at high risk of SNA, which was further found to be associated with multiple negative health consequences including poor sleeping quality, mental well-being, and academic performance. This suggests that SNA has become a serious public health issue affecting the lives of a considerable number of young adults. Effective intervention and prevention strategies are critically needed to tackle the problem in a timely manner. Meanwhile, parental reactive restriction and limiting online behavior were associated with higher probability of university students' SNA.

We found an overall SNA prevalence of 21.5% among the surveyed Hong Kong university students, which is consistent with previous findings showing that 12–34% of young adults are problematic users of SNSs worldwide (10, 70). Nonetheless, while some evidence suggests that SNA appears to be more prevalent among females (27), the present study found SNA to be unrelated to university students' gender. A recent report based on college students in Singapore showed similar findings (21). It should be noted that despite the non-significant gender difference in prevalence, males and females may use SNSs for distinct purposes and display different online behaviors associated with SNS usage (71). For example, Muscanell and Guadagno (72) reported that men tended to use SNSs for forming new relationships while women were more likely to use SNSs for relationship maintenance. Females also reported using SNSs more broadly than did males (73). Further investigation into gender-specific motivations and SNS usage behaviors would help inform the development of prevention and intervention programs targeting different gender groups.

It is worth noting that the most frequently reported SNA behaviors in the present study are related to “mood modification,” “salience,” and “withdrawal symptoms.” More than 40% of the participating students indicated that they used SNSs to escape from negative feelings, felt bad when they could not use SNSs, and tried to spend less time on SNSs but failed. These behaviors resemble some core symptoms of other types of behavioral addiction like gambling and gaming disorder (74). Such problematic behavioral patterns often lead to further emotional, performance-related, and health problems in one's life.

In fact, we found that students with SNA in the present study reported poorer sleeping quality (longer sleep latency and more sleep disturbance), lower levels of life satisfaction and perceived academic performance, and higher levels of depression. These findings provide further evidence for the pervasive negative impact of SNA on the health and well-being of university students in the context of Hong Kong. Corroborating with prior reports based on other populations (75–77), the present study suggests that despite of the positive benefits and impacts of SNS, there is a significant detrimental effect on many aspects of life including academic achievement among people who cannot control their excessive uses of SNS, especially youth who are still in education. Recent studies showed that cognitive reconstruction about SNSs use could effectively mitigate SNA by helping students realize the adverse effects of SNA and the potential benefits of reducing SNS usage (78). The present findings can be used in the development of such psychoeducational programs to prevent and treat SNA among university students in Hong Kong. Besides, intervention programs targeting SNA shall not only focus on reducing one's SNS use, but develop strategies to improve the health and well-being status of the addictive users, such as addressing their depression and sleeping problems, and providing support to improve their learning efficiency and academic performance.

Regarding the influence of parental Internet-specific behaviors, the present study revealed a positive relationship between parental restrictive mediation (i.e., reactive restriction and limiting online behaviors) and university students' SNA. In other words, more controlling behaviors displayed by parents regarding their children's use of SNSs (e.g., asking their children to turn off their mobile phones) are associated with a higher risk of SNA among university students. This counterintuitive finding may actually reflect a reverse causality, i.e., parents simply engage in more restrictive behaviors after observing their children's addictive use of SNSs. Previous studies showed inconsistent findings where parental restrictive mediation was found to be positively (79, 80), negatively (41, 81), or insignificantly (82) related to their children's addictive use of Internet. Researchers have proposed that the association between parental mediation strategies and their children's behavior may be further moderated by other factors (37, 82–84). For example, Lee (82) reported the child's low self-control reinforced the effects of restrictive mediation on the child's online time. Besides, the effects of concrete parenting practices (i.e., Internet-specific parenting behaviors) may also be moderated by general family functioning and parenting style (83). Parent-child relationship and family dynamics have consistently been found to affect the influence of specific parenting practices on children's development and mental health (85, 86). In the present study, we did not include indicators of general parenting behaviors (e.g., responsiveness and demandingness) or family climate when investigating the influence of Internet-specific parental behaviors on university students' SNA. Such indicators shall be taken into account in future studies. More importantly, the causal relationship between Internet-specific parenting behavior and children's SNA, as well as the potential moderators must be addressed based on longitudinal research.

Other parenting behaviors including parents' own SNS usage, social co-use, and quality of communication were all found to have no significant relationship with university students' SNA. This is consistent with existing findings showing that the salience of parents' direct influence on adolescents' behaviors recedes as adolescents increasingly incorporate more extra-familial socialization influence into their sense of identity (87, 88). Peer modeling and norms have been highlighted as powerful determinants of young people's addictive behaviors such as drinking and digital game addiction (89, 90). Besides, although most university students in Hong Kong remain living at home, they spend little time with their parents. A recent survey revealed that 92% of Hong Kong university students had extra-curricular jobs for further financial support (91), which likely took away from family time. Limited family time may further weaken parental influence on children's behaviors. In addition, similar to parental restrictive mediation, the effects of Internet-specific parenting behaviors (e.g., parents monitoring children's SNS use) are likely to be moderated by one's family relationships and the general parenting styles that parents display over time. We need more in-depth investigation into the role of family dynamics in the association between parenting behaviors and young people's SNA.

Several limitations of the present study must be acknowledged. First, although we found significant relationships between SNA and different health indicators, the direction of the relationships cannot be confirmed based on the cross-sectional study. Some scholars (25, 92) have reported reciprocal relationships between Internet addiction and one's well-being, indicating the possibility that individuals with more emotional disturbance may be more likely to use SNSs intensively in order to escape from the real world, and the excessive time used on SNSs further causes problems in one's life. This issue must be addressed with multiple waves of data collected in future longitudinal research. Second, parenting behaviors were measured only based on university students' perceptions. Further studies should collect data from multiple informants, for example by using parents' self-reported behaviors. Third, participants in the present study were recruited from a convenient sample. To gain a more accurate estimation of the prevalence rate of SNA among university students in Hong Kong, large-scale studies based on representative samples are necessary.

Despite these limitations, the present study is among the first to examine SNA and its relationships with multiple health outcomes and parenting behaviors among university students in Hong Kong. The findings highlight the severity of the issue. Timely prevention and detection of SNA should be given priority in terms of health education resources for university students. Moreover, as our understanding of parenting behaviors regarding SNS usage continues to develop, researchers in this area might be able to provide a more complete and clear framework of the relationship between SNA and parenting behaviors, as well as the underlying mechanism.

## Data Availability Statement

The raw data supporting the conclusions of this article will be made available by the authors, without undue reservation.

## Ethics Statement

The studies involving human participants were reviewed and approved by Human Subjects Ethics Sub-committee, Department of Applied Social Sciences, The Hong Kong Polytechnic University. The patients/participants provided their written informed consent to participate in this study.

## Author Contributions

LY conceptualized and designed the study, collected the data, interpreted the data, drafted the manuscript, and approved the final manuscript as submitted. TL collected the data, interpreted the data, drafted the manuscript, and approved the final manuscript as submitted. Both authors contributed to the article and approved the submitted version.

## Conflict of Interest

The authors declare that the research was conducted in the absence of any commercial or financial relationships that could be construed as a potential conflict of interest.
